# Hsp90/Cdc37 Chaperone/co-chaperone complex, a novel junction anticancer target elucidated by the mode of action of herbal drug Withaferin A

**DOI:** 10.1186/1471-2105-12-S1-S30

**Published:** 2011-02-15

**Authors:** Abhinav Grover, Ashutosh Shandilya, Vibhuti Agrawal, Piyush Pratik, Divya Bhasme, Virendra S Bisaria, Durai Sundar

**Affiliations:** 1 Department of Biochemical Engineering and Biotechnology, Indian Institute of Technology (IIT) Delhi, Hauz Khas, New Delhi 110016, India; 2 Supercomputing Facility for Bioinformatics and Computational Biology, Indian Institute of Technology (IIT) Delhi, Hauz Khas, New Delhi 110016, India

## Abstract

**Background:**

HSPs (Heat shock proteins) are highly conserved ubiquitous proteins among species which are involved in maintaining appropriate folding and conformation of other proteins and are thus referred to as molecular chaperones. Hsp90 (Heat-shock protein 90 kDa) is one of a group of molecular chaperones responsible for managing protein folding and quality control in cell environment. However it is also involved in the maturation and stabilization of a wide range of oncogenic client proteins which are crucial for oncogenesis and malignant progression. Hsp90 requires a series of co-chaperones to assemble into a super-chaperone complex for its function. These co-chaperones bind and leave the complex at various stages to regulate the chaperoning process. Arresting the chaperone cycle at these stages by targeting different co-chaperone/Hsp90 interactions seems to be quite a viable alternative and is likely to achieve similar consequences as that of Hsp90 direct inhibition with added favors of high specificity and reduced side effect profile. The study conducted here is an attempt to explore the potential of *Withania somnifera’s* major constituent WA (Withaferin A) in attenuating the Hsp90/Cdc37 chaperone/co-chaperone interactions for enhanced tumor arresting activity and to elucidate the underlying mode of action using computational approaches.

**Results:**

Formation of active Hsp90/Cdc37 complex is one of the essential steps for facilitation of chaperone client interaction, non-assembly of which can lead to prevention of the chaperone-client association resulting in apoptosis of tumor cells. From our flexible docking analysis of WA into active Hsp90/Cdc37 complex in which key interfacing residues of the complex were kept flexible, disruption of the active association complex can be discerned. While docking of WA into segregated Hsp90 leaves the interface residues untouched. Thus the molecular docking analysis of WA into Hsp90 and active Hsp90/Cdc37 complex conducted in this study provides significant evidence in support of the proposed mechanism of chaperone assembly suppression by inhibition or disruption of active Hsp90/Cdc37 complex formation being accounted by non-assembly of the catalytically active Hsp90/Cdc37 complex. Results from the molecular dynamics simulations in water show that the trajectories of the protein complexed with ligand WA are stable over a considerably long time period of 4 ns, with the energies of the complex being lowered in comparison to the un-docked association complex, suggesting the thermodynamic stability of WA complexed Hsp90/Cdc37.

**Conclusions:**

The molecular chaperone Hsp90 has been a promising target for cancer therapy. Cancer is a disease marked by genetic instability. Thus specific inhibition of individual proteins or signalling pathways holds a great potential for subversion of this genetic plasticity of cancers. This study is a step forward in this direction. Our computational analysis provided a rationalization to the ability of naturally occurring WA to alter the chaperone signalling pathway. The large value of binding energy involved in binding of WA to the active Hsp90/Cdc37 complex consolidates the thermodynamic stability of the binding. Our docking results obtained substantiate the hypothesis that WA has the potential to inhibit the association of chaperone (Hsp90) to its co-chaperone (Cdc37) by disrupting the stability of attachment of Hsp90 to Cdc37. Conclusively our results strongly suggest that withaferin A is a potent anticancer agent as ascertained by its potent Hsp90-client modulating capability.

## Background

Heat shock proteins (HSPs) are highly conserved ubiquitous proteins among species which are involved in maintaining appropriate folding and conformation of other proteins and are thus referred to as molecular chaperones [1]. Hsp90 (90 kDa heat-shock protein) is one of a group of molecular chaperones responsible for managing protein folding and quality control in cell environment [2]. It is one of the most abundant proteins in the eukaryotic cells comprising 1-2% of total proteins under non-stress conditions [3]. Although Hsp90 is involved in assisting protein folding and preventing aggregation of non-native proteins, it also plays a key role under normal conditions in regulating the stability and activation state of more than 200 ‘client’ proteins molecules including transmembrane tyrosine kinasestastable signaling proteins (Akt, Raf-1 and IKK), mutated signaling proteins (p53, Kit, Flt3 and v-src), chimeric signaling proteins (NPM–ALK, Bcr–Abl), steroid receptors (androgen, estrogen and progesterone receptors) and cell-cycle regulators (cdk4, cdk6) [4]. Recent systems-biology studies indicate that Hsp90 is a major interaction node, regulating a very diverse set of cellular functions [5-7].

### Hsp90 as a therapeutic target in cancer

Hsp90 is involved in the maturation and stabilization of a wide range of oncogenic client proteins which are crucial for oncogenesis and malignant progression [8], the cancer cells being particularly dependent on proper Hsp90 function [9,10]. Many of these client proteins are mutated and/or overexpressed in cancers [11]. Moreover, the harsh environmental conditions found in tumors such as hypoxia, low pH, and bad nutritional status may tend to destabilize proteins, making them even more dependent on Hsp90 activity [8,12,13]. It has been observed that Hsp90 is constitutively expressed at manifold levels in tumor cells compared to their normal counterparts [14], suggesting that Hsp90 is critically important for tumor cell growth and and/or survival and its inhibition would help check the proliferation of cancer cells. Inhibition of Hsp90 would lead to the proteasomal degradation of a large number of oncogenic client proteins, offering an additional favour of combinatorial impact on multiple oncogenic pathways and amelioration of all the cancer attributing traits, including proliferation, evasion of apoptosis, immortalization, invasion, angiogenesis and metastasis [9]. In addition, this combinatorial action markedly reduces the opportunities for cancer cells to develop resistance to Hsp90 inhibition [15]. The association of Hsp90 with a plethora of pathways including signal transduction, cell-cycle control, transcriptional regulation and many others [16-18] marks it as target for pharmacological modulation in a number of diseases, particularly for cancer, where the chaperoning of mutated and overexpressed oncoproteins is critical [19]. This has led to the development of Hsp90 inhibitors for treatment of human cancers [4,20].

### Development of Hsp90 inhibitors

Chaperoning of Hsp90 client proteins is regulated through a dynamic cycle driven by ATP binding to Hsp90 and subsequent hydrolysis [21]. Thus blocking of ATP binding has developed as a single well known mechanism for development of Hsp90 inhibitors. The currently established natural product inhibitors of Hsp90 like radicicol, benzoquinone ansamycins like geldanamycin and its derivative 17-allylamino geldanamycin, which are all based on the same ATP blocking criterion, cause the catalytic cycle of Hsp90 to arrest in the ADP-bound conformation, leading to the inactivation of chaperone activity, premature ubiquitination and of client proteins [22,23]. The proteasomal degradation of the client proteins finally result in depletion of oncoproteins, cell cycle arrest and consequent apoptosis [24]. Though blockade of ATP binding seems to be the most direct and the simplest way to inhibit Hsp90 function, the intrinsic non-selectivity of this method for Hsp90 clientele limits its further application [25]. Of all the inhibitors employing ATP blockage mechanism for Hsp90 inhibition, none has received the Food and Drug Administration approval [26], providing space for exploration of alternative inhibition mechanisms.

### Blocking of co-chaperone/chaperone interactions

It has been well reported that Hsp90 requires a series of co-chaperones to assemble into a super-chaperone complex for its function. These co-chaperones bind and leave the complex at various stages to regulate the chaperoning process [21]. Arresting the chaperone cycle at these stages by targeting different co-chaperone/Hsp90 interactions seems to be quite a viable alternative and is likely to achieve similar consequences as that of Hsp90 direct inhibition [26,27]. Cdc37 (Cell division cycle protein 37), one of the essential co-chaperone of Hsp90, plays a specialized and indispensable role in the maturation of a number of kinase clients, whose functions are commonly dysregulated in cancer, such as receptor tyrosine kinases like epidermal growth factor receptor (EGFR), non-receptor tyrosine kinases Src and lymphocyte specific protein tyrosine kinase (Lck), and intracellular serine/threonine kinases such as Raf-1and Cdk4 [28]. Cdc37 facilitates the maturation of these kinase clients by acting as an adaptor, loading these kinases onto the Hsp90 complex [29-31]. Knocking out of Cdc37 using RNAi has been shown to attenuate the kinase clients’ association to Hsp90 in human colon cancer cells, leading to reduced cell proliferation. Furthermore, combining Cdc37 silencing with the HSP90 inhibitor 17-AAG induced more extensive and sustained depletion of kinase clients and potentiated cell cycle arrest and apoptosis. These results support an essential role for Cdc37 in concert with Hsp90 in maintaining oncogenic protein kinase clients and endorse the therapeutic potential of targeting Cdc37/Hsp90 in cancer [32].

Targeting Cdc37/Hsp90 interaction represents a potential alternative to direct Hsp90 inhibition that may offer greater specificity (due to Cdc37 elevated expression in cancer) and an improved side effect profile [27]. Additionally, Cdc37 may be an attractive target in tumor types such as androgen independent prostate cancers that currently lack highly effective therapies. Recently, Celastrol, a quinone methide triterpene from *Tripterygium wilfordii* , has been shown to exhibit anti-pancreatic cancer activity both *in vitro* and *in vivo* through the disruption of Cdc37/Hsp90 association and the subsequent degradation of Hsp90 client proteins [26].

**Figure 1:**
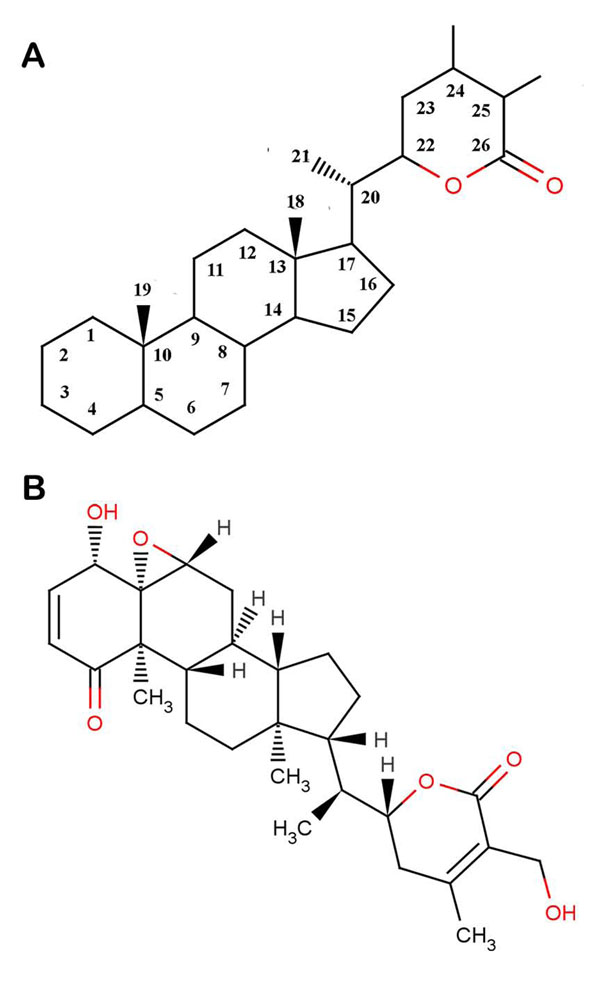
**Structures of withanolides.** (A) Withaferin A falls under the family of compounds known as withanolides which are a group of naturally occurring C28- steroidal lactones built on an intact or rearranged ergostane framework, in which C-22 and C- 26 are appropriately oxidized to form a six-membered lactone ring. The basic structure is designated as the withanolide skeleton defined as a 22-hydroxyergostan-26-oic acid-26,22-lactone. (B) Structure of withaferin A

*Withania somnifera*, also known as “ashwagandha”, “Queen of Ayurveda”, “Indian ginseng”, and “winter cherry”, has been an important herb in the Ayurvedic and indigenous medical systems for more than 3,000 years [33]. Its roots have been used as herb remedy to treat a variety of ailments and to promote general wellness. It has received much attention in recent years due to the presence of a large number of alkaloids and steroidal lactones known as withanolides [34,35]. The structure of basic skeleton of withanolides is shown in Figure 1. The principal withanolide present in the plant is WA which is known to have anti-inflammatory [36], antitumor [37], antibacterial [38], antioxidant [39], anticonvulsive [40], and immunosuppressive properties [41]. It has the potential to increase tumor sensitization to radiation and chemotherapy while reducing some of the most common side effects of these conventional therapies [42]. Most recently, WA has been shown to potentiate apoptosis of tumor cells by inhibiting Hsp90 in human pancreatic cancer cells [43], protect against UV-induced skin cancer [44] and enhance neurite regeneration and memory [45,46]. Thus, many studies have been reported depicting the effect of WA on putting a check on human cancers, but the mechanism behind this effect is still eluding the researchers. The study conducted here is an attempt to explore the potential of *Withania somnifera’s* major constituent WA in attenuating the Hsp90/Cdc37 chaperone/co-chaperone interactions for enhanced tumor arresting activity and to elucidate the underlying mode of action using computational approaches.

## Methods

### Ligand and Receptors

The crystal structure of the Hsp90/Cdc37 association domain [PDB: 2K5B] was obtained from the Protein Data Bank (PDB) [47]. The crystal structure contained many missing atoms which were supplemented by the repairCommands module of AutoDock. Before docking, the protein crystal structure was cleaned by removing the water molecules. H-atoms were added to these target proteins for correct ionization and tautomeric states of amino acid residues. The modified structure so obtained was used for all the flexible docking studies while only Hsp90 [PDB: 3HZ5] was used for performing semi-flexible dockings. The ligand molecule withaferin A [PubChem:265237] was retrieved from NCBI-PubChem Compound database [48]. The structure of this compound is shown in Figure 1. The energy of the ligand molecule and receptors were minimized in Steepest Descent and Conjugate Gradient methods using Amber 11 [49], the most comprehensive suite for carrying molecular dynamics simulations.

### Structural aspects of Cdc37-Hsp90 complex

Hsp90 is composed of three domains: an N-terminal domain of ~25-kDa, a middle domain of ~35-kDa, and a C-terminal domain of ~10-kDa [50]. The ATP-binding site of Hsp90 is located in the N-terminal domain. The 44.5-kDa Cdc37 protein can be dissected into three domains [51,52] : an N-terminal domain (residues 1-127 (Cdc37_N_), 15.5 kDa), a middle domain (residues 147- 276 (Cdc37_M_) 16 kDa), and a C-terminal domain (residues 283-378 (Cdc37_C_), 10.5 kDa). The middle domain Cdc37_M_ is highly resistant to proteolytic digestion and was found to be the most stable domain of Cdc37 [53]. The Hsp90 binding site is located in the middle domain of Cdc37 which associates with the Nterminal domain of Hsp90 [54,55]. The structural features of the receptor macromolecule [PDB: 2K5B] have been described in detail elsewhere [47] by the depositors of the crystal structure to the Protein Data Bank. Briefly, the receptor is an association complex of Cdc37_M_ and Hsp90_N_ in respectively two chains. Hsp90_N_ density extends from residues Glu 14 to Glu 223 in chain A and the Cdc37_M_ peptide density extends from residues Lys 148 to His 276 in chain B. The Cdc37 peptides are almost helical except for few unwound stretches ranging from 164–168. For Hsp90, residues 117, 121, 123, 124, 125, 126, and 129 have been defined as active. For Cdc37_M_, residues 160, 161, 164, 165, 166, 167, 168, 193, 202, 204, 205, and 208 have been defined as Active. The Cdc37_M_ residues that appear to be on the interface region of the association complex include Lys-160, Met-164, Arg-166, Arg-167, Trp-168, Leu-205, Glu-207, Gln-208, and Ile-214. Electrostatic interactions are observed between the residues Arg-166, Arg-167, and Asp-170 of Cdc37_M_ and Gln-133 of Hsp90_N_. Arg-166 and Arg-167 residues of Cdc37 peptide form intermolecular hydrogen bonds with residue Gln-133 of Hsp90_N_. The hydrophobic core involves residues Met-164, Trp-193, Ala-204, and Leu-205 of Cdc37_M_ and Ala-117, Ala-121, Ala-124, Ala-126, Met-130, and Phe-134 of Hsp90_N_. The side chain of Glu-120 of human Hsp90 forms a hydrogen bond with the Lys-202 side chain of Cdc37. An important hydrogen bond is formed between Glu-47 of Hsp90 and Arg-167 of Cdc37, which is considered to be the key interaction that arrests the ATPase action of Hsp90 [54]. It was also observed that the amino moiety of Gln 208 of Cdc37 is rather close to the side-chain hydroxyl group of the Ser-113 residue of Hsp90, with which it may possibly form an intermolecular hydrogen bond.

### Semi-flexible docking

AutoDock 4.0 suite was used as molecular-docking tool in order to carry out the docking simulations [56]. AutoDock has been found to be able to locate docking modes that are consistent with X-ray crystal structures [57,58]. AutoDock helps to simulate interactions between substrates or drug candidates as ligands and their macromolecular receptors of known three dimensional structures, allowing ligand flexibility described to a full extent elsewhere [56]. In our docking simulations we first used only the human HSP90 X-ray crystal structure [PDB: 3HZ5] for performing semi-flexible docking, with the ligand WA made flexible while keeping the receptor macromolecule being rigid. Flexibility of the ligand helps it explore six spatial degrees of freedom for rotation and translation and an arbitrary number of torsional degrees of freedom. A random perturbation to each is applied at each time step, and the interaction energy was evaluated for the new location and conformation [59].

### Flexible docking

One of the novel features of AutoDock 4.0 allows side chains in the protein as well as in the ligand to be flexible. Thus flexibility of receptor molecule was also exploited in docking studies by making use of AutoDock flexres scripts. It is worth assumable that the incoming ligand arriving at the binding sites of the two chains would try to make its own associations with the residues in order to minimize the energy of the system. To facilitate this notion, the key residues of Hsp90 and Cdc37 chains, majority of which form H-bonds with corresponding residues in the two chains as reported [47], were made flexible in order to observe their mode of interactions with the ligand and to account for the subsequent rearrangements. The peptide bonds of the amino acids chosen to be flexible were kept “inactive” or non-rotatable. The Graphical User Interface program "AutoDock Tools" was used to prepare, run, and analyze the docking simulations. Protein was prepared for docking simulations by assigning of Kollman united atom charges, solvation parameters and polar hydrogens to the receptor PDB file. Water molecules were removed from its PDB file to make it a free receptor. Since ligands are not peptides, Gasteiger charge was assigned and then nonpolar hydrogens were merged. AutoDock assigns the rigid roots to the ligand automatically saving time as compared to manual picking. Five bonds in the ligand were made “active” or rotatable. Atomic solvation parameters were assigned to the receptor using default parameters.

### Grid design

Auto-Tors, an auxiliary program using Interactive queries to define rotatable torsion angles, is used to assign all rotatable dihedrals in the ligands and to remove non-polar hydrogen atoms, uniting their partial charges with their bonded carbon atoms. AutoGrid, which calculates grids of interaction energy based on the interaction of the ligand atom probes with receptor target, is used to obtain the grid maps required prior to docking. Each probe consists of an atom type present in the ligand being docked. The pre-calculated grid maps store the potential energy arising from the interaction with the macromolecule. The user defined three dimensional grid must surround the region of interest in the macromolecule, and the ligand was limited to this search space during docking. In the present study, the location and dimensions of the grid box are chosen such that it incorporates the flexible residues which are involved in binding of Cdc37 to Hsp90 for the formation of active complex. The energy scoring grid was prepared as a 36, 30 and 40 A° (x, y, and z) cube for semi-flexible docking and 60, 60, and 60 A° (x, y, and z) cube for flexible docking. The spacing between grid points was 0.375 angstroms.

### The Genetic Algorithm

The Lamarckian Genetic Algorithm (LGA) was chosen to search for the best conformers. Using mathematical concepts designed to simulate the conditions influencing biological evolution, genetic algorithms are able to search conformational space by "mutating" a ligand in order to find its lowest energy conformation in the "environment" of a fixed protein. Searches driven by this energy funnelling have been shown to provide a good indication as to the optimum protein-ligand interactions and therefore conveying the structure most likely to be found *in vivo*[60]. The default parameters for the Lamarckian genetic algorithm [56] were used as the search protocol except for the maximum number of energy evaluations, which were changed to 2.5 million. During the docking process, a maximum of 10 conformers was considered for each compound. The population size was set to 150 and the individuals were initialized randomly. Maximum number of generations was kept as 15000, maximum number of top individuals that automatically survived set to 1 with a mutation rate of 0.02 and a crossover rate of 0.8. Step sizes for translations, quaternions and torsions were kept same as defaults. An efficient and accurate energy assessment of the ligand conformations is as important to the success of a docking simulation as the power of the search algorithm. AutoDock uses a variation on the '95 force field [49] with terms empirically determined by linear regression analysis from a set of protein-ligand complexes with known binding constants [56,61]. Gibbs Free energy (ΔG) is calculated as a sum of six energy terms of dispersion/repulsion, hydrogen bonding, electrostatic interactions, deviation from covalent geometry, internal ligand torsional constraints, and desolvation effects.

### Selection and representation of docking modes

AutoDock reports the best docking solution (lowest docked free energy) for each GA run and also performs a cluster analysis in which the total number of clusters and the rank of each docking mode (cluster rank) are reported. Docking modes were selected on the basis of two criteria: ligand’s proximity to the Asp 166 of middle Cdc37 chain and extent of the ligand’s associations with the Cdc37-Hsp90 interacting residues. For a 10 GA run there would be up to 10 total docking modes from which the lowest energy-docking mode was chosen that met the above two criteria. All the AutoDock docking runs were performed in Intel Core 2 Duo P8400 CPU @ 2.26 GHz of Sony origin, with 3 GB DDR RAM. AutoDock 4.0 was compiled and run under Windows VISTA operating system. The output from AutoDock and all modeling studies as well as images were rendered with PyMOL [62] and Accelerys ViewerLite 5.0. ViewerLite was used to calculate the distances of hydrogen bonds as measured between the hydrogen and its assumed binding partner.

### Confirmation of the docking results

The docking results obtained using AutoDock were also confirmed using ParDOCK [63] , which is an all atom energy based monte carlo docking protocol. Docking using ParDOCK requires a reference complex (target protein bound to a reference ligand) and a candidate molecule along with specific mention of the centre of mass of the cavity on which the ligand is to be docked.

### MD simulations in water

The AMBER v.11 package [64] was used to prepare the protein and the ligand files as well as for the Molecular Dynamics (MD) simulations. The binding complexes of Hsp90/Cdc37/WA and Hsp90/WA obtained using ParDOCK, and the un-docked proteins simulated in this study were neutralized by adding appropriate number of sodium counter-ions and were solvated in a octahedron box of TIP4PEW water with a 10 Å distance between the protein surface and the box boundary [65]. The partial atomic charges for the ligand were obtained using “antechamber” [66] module of Amber.

The energy minimization and MD simulations of Hsp90, Hsp90/Cdc37 and their complex with WA were carried out with the aid of the SANDER module of the AMBER 11 program. First of all, the simulated binding complex was effected with a 750 step minimization using the steepest descent algorithm followed by a 250 step minimization using conjugate gradient to remove bad steric contacts. Topology and parameter files for the protein were generated using “ff99SB” and for the drug using “gaff” based on the atom types of the force field model developed by Cornell et al [49]. Then the system was equilibrated beginning with the protein atom restrained simulations having 200 ps equilibration dynamics of the solvent molecules at 300 K and a harmonic potential with a 10 kcal/mol restraint force. Next step involved the equilibration of the solute molecules with a fixed configuration of the solvent molecules in which the system was slowly heated from T = 0 to 300 K in 60 intervals each involving heating for a 5 K increase in 2.5 ps followed by an equal time duration equilibration step. The entire system was then equilibrated at 300 K for 200 ps before a sufficiently long MD simulation (for 4 ns) at room temperature. The MD simulations were performed with a periodic boundary condition in the NPT ensemble at T=298.15 K with Berendsen temperature coupling [67] and constant pressure P=1 atm with isotropic molecule-based scaling . The SHAKE algorithm [68] was applied to fix all covalent bonds containing hydrogen atoms. We used a time step of 2 fs and a nonbond-interaction cutoff radius of 10 A°. The Particle Mesh Ewald (PME) method [69] was used to treat long-range electrostatic interactions. The coordinates of the trajectory was sampled every 2.5 ps for analysis of the energy stabilization and RMSD values of the protein as well as that of the complex. MD simulations were performed on a 320 processors SUN Microsystems clusters at Supercomputing Facility (SCFBio) at IIT Delhi.

## Results and discussion

### Semi-flexible docking of WA into H90

**Figure 2:**
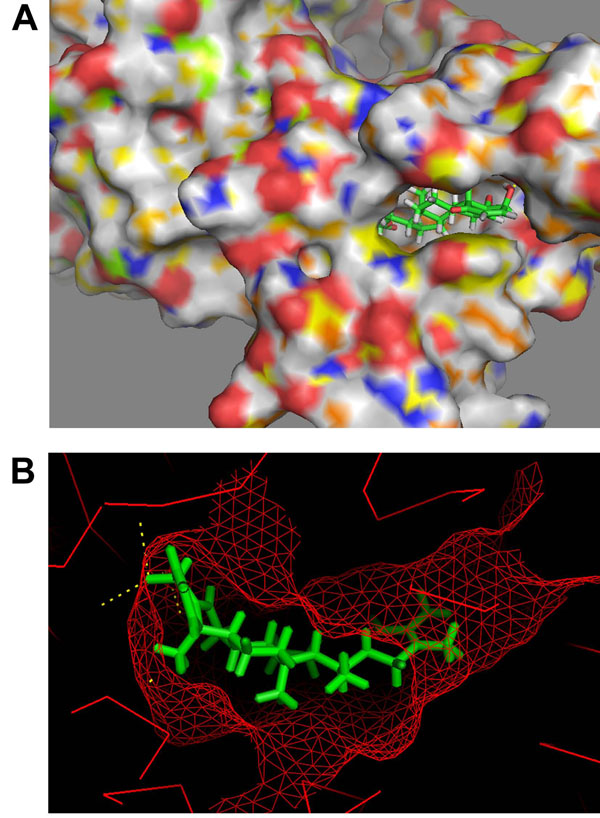
**Docking representations of Withaferin A.** (A) Ligand docked into the Hsp90 receptor cavity (B) Docked Ligand inside the pocket of Hsp90 receptor mesh

**Figure 3:**
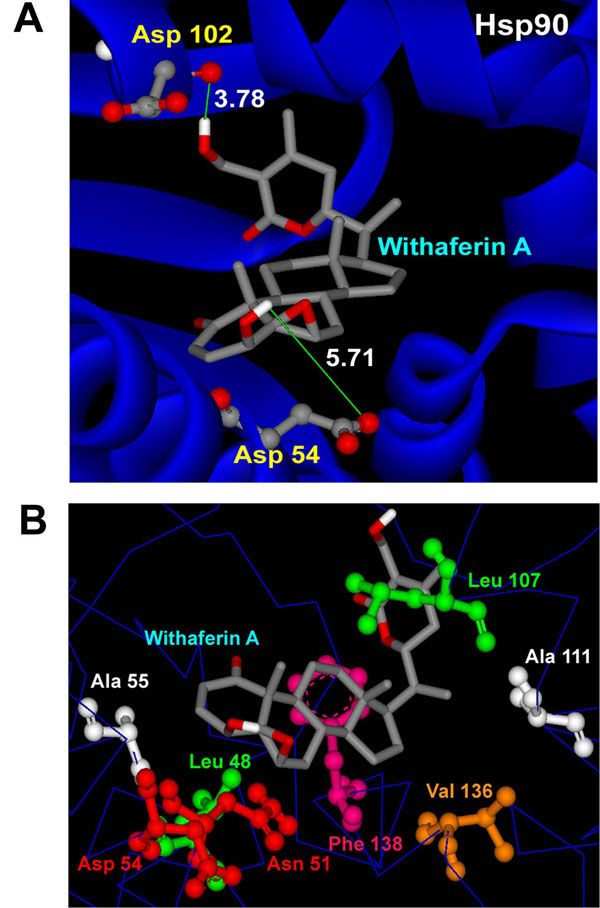
**Interactions of docked withaferin A with Hsp90 receptor.** (A) H-Bond interactions of the docked ligand with Hsp90 residues. (B) Docked withaferin A forming vdw interactions with the hydrophobic residues of Hsp90.

**Figure 4:**
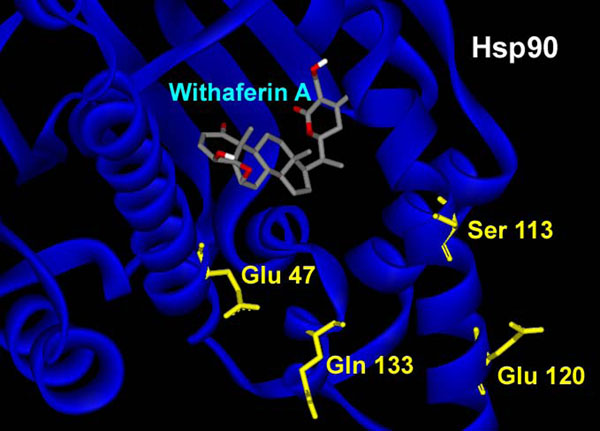
**Interactions of docked withaferin A with Hsp90 receptor.** The docked withaferin A does not interact with any of the Cdc37 interacting residues of Hsp90.

One possible mode of action which is proposed here for WA to act as a chaperone function inhibitor is by restriction/disintegration of the complex between Hsp90 and Cdc37. In order to explore the possibility of non-formation of the complex, we first carried out molecular docking studies with only the Hsp90 protein crystal structure. Before docking to be carried out, the structures of receptor macromolecules were minimized using Sander module of Amber. Macromolecular receptors minimized using Steepest Descent and Conjugate Gradient methods had comparatively lower potential energy values than those of the initial ones and are thus utilized further for carrying out the docking studies. Figure 2A shows the docked ligand WA into the selective HSP90 receptor. WA gets buried inside the pocket of HSP90 as depicted by mesh representation in Figure 2B. For this particular configuration the binding energy of WA with Hsp90 is -9.10 Kcal/mol with an Inhibition constant of 214.73 nM (Table 1). The binding of WA to Hsp90 is characterized by H-bonding between a terminal hydroxyl group of the ligand and the side chain carboxyl group of Asp 102. The other terminal hydroxyl group of WA is also involved in a weak H-bond with Asp 54 of Hsp90 (Figure 3A). The lengths of the two H-bonds formed are 3.78 and 5.71 Å. In the present docked structure, WA is also forming van der waals interactions with Leu 48, Asn 51, Asp 54, Ala 55, Leu 107, Ala 111, Val 136 and Phe 138 of Hsp90 (Figure 3B). These non-covalent interactions help stabilize the binding of the ligand with the macromolecule by lowering the energy. However, no interaction of WA with the proposed Cdc37 interacting residues could be observed, as is evident from Figure 4. These finding suggests that binding of WA to Hsp90 could only provide a naive hinderance to the attachment of Cdc37 to Hsp90, owing to the presence of yet available Cdc37 interacting residues of Hsp 90 upon WA binding. However, WA binding to Hsp90 may result in the formation of a deformed complex as the Hsp90/Cdc37 complex would not get assembled to its catalytically active form.

**Table 1:** Energies obtained after docking of withaferin A into Hsp90

Property	Quantity
Binding Energy	-9.10 Kcal/mol
Inhibition constant	214.73 nM
Intermolecular energy	-10.36 Kcal/mol
Total internal energy	-0.11 Kcal/mol

### Flexible docking of WA into active Hsp90/Cdc37 complex

**Figure 5:**
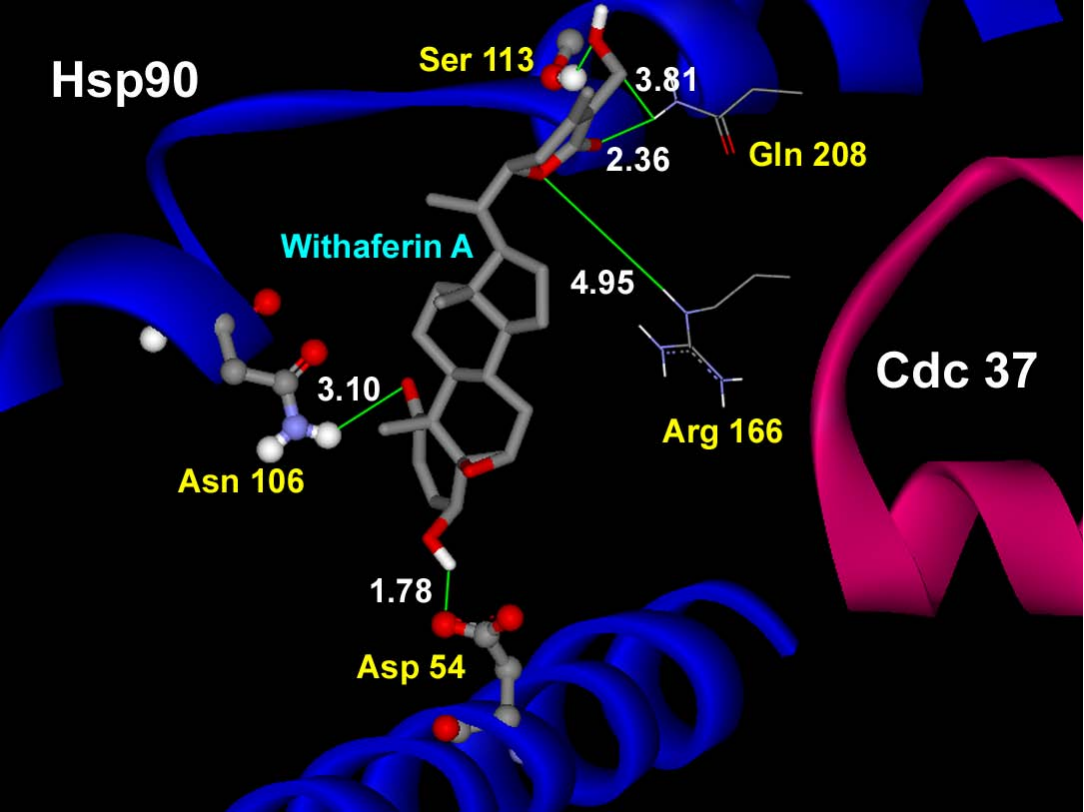
**H-bonding interactions present in the docked complex of withaferin A with active Hsp90/Cdc37 complex using flexible docking.** Polar hydroxyl terminal group of withaferin A captivates amino group of Gln 208 of Cdc37 to form H-bond with itself, thus disrupting the earlier present Gln 208 - Ser 113H-bond. WA also disrupts the H-bond between Gln 133-Arg 166 by forming H-bond with Arg 166 of Cdc37. WA also forms H-bonds with Asp 54 and Asp 106 of Hsp90.

Docking of WA performed on the catalytically active human Hsp90/Cdc37 complex provided interesting results. The binding of WA to the association complex provides significant evidence in support of the proposed mechanism of chaperone/co-chaperone activation suppression by inhibition or disruption of active Hsp90/Cdc37 complex. Two clusters were obtained for Genetic Algorithm run from generation of ten models. Large negative binding energies were obtained for both the clusters as is evident from Table 2. The lowest binding energy (-13.95 Kcal/mol) was much lower than that obtained obtained from binding of WA to the segregated Hsp90 receptor. As shown in Figure 5, the hydroxyl group of WA disrupts Ser 133:Gln 208 H-bond present in the active complex by itself hydrogen bonding with both Ser 133 . One of the ring carbonyl group of the ligand is involved in H-bonding with Gln 208 also, which has been reported as one of the critical residues in complex formation [47]. The same ring lactone group forms H-bond with Arg 166 residue of Cdc 37. Arg 166 and Arg 167 residues of Cdc37 are involved in hydrogen bonding with Gln 133 of Hsp90 in the original functional complex. Thus from our flexible docking results, it is clear that the binding of WA to the interface of Hsp90/Cdc37 structurally disrupts the active complex. The absence of active chaperone/co-chaperone complex would prevent catalysis of the conformational maturation of the Hsp90 client proteins. This would ultimately lead to degradation of Hsp90 clients thus arresting their nefarious acts.

**Table 2:** Clustering results obtained from docking of withaferin A into Hsp90/Cdc37 complex

Receptor	**No. of AutoDock clusters**^**a, b**^	**Cluster rank**^**b**^	No. of structures in the cluster	Lowest binding energy of cluster	Energy range within cluster
		1	3	-13.95	-13.95 to -10.18
Hsp90/Cdc37 complex	2 (10)	2	7	-13.58	-13.58 to -10.11

^a^ Number of GA runs are shown in parentheses

^b^ Clustering is done with RMS tolerance of 5.0 Å

The results obtained from docking of WA into the active complex, with a majority of earlier reported H-bond and hydrophobic interaction forming residues kept flexible, clearly show that WA blocks the intermolecular interactions between Hsp90 and Cdc37 at the residues which are significantly involved in formation of the active complex. The large value of binding energy (-13.95 Kcal/mol) involved in binding of WA to the complex consolidates the thermodynamic stability of the binding. These results substantiate the hypothesis that WA possess the potential to disarray the active complex by disrupting the stability of attachment of Hsp90 and Cdc37 chains, being accounted by hydrophobic and H-bond interactions.

### MD simulations in water

**Figure 6:**
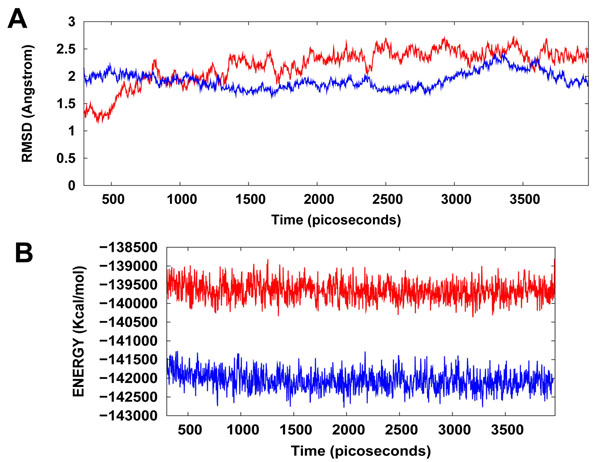
**(A) Plot of root mean square deviation (RMSD) of Cα of Hsp90/Cdc37 (protein) and Hsp90/Cdc37/WA (complex).** RMSDs were calculated using the initial structures as templates. For protein (red) the reference is the PDB structure and for complex (blue) the reference is the initial docked structure. The trajectories were captured every 2.5 ps until the simulation time reached 4000 ps. **(B) Plot of total energy of Hsp90/Cdc37 and Hsp90/Cdc37/WA (complex)** The energy trajectories of both the protein (red) and the complex (blue) are stable over the entire length of simulation time.

**Figure 7:**
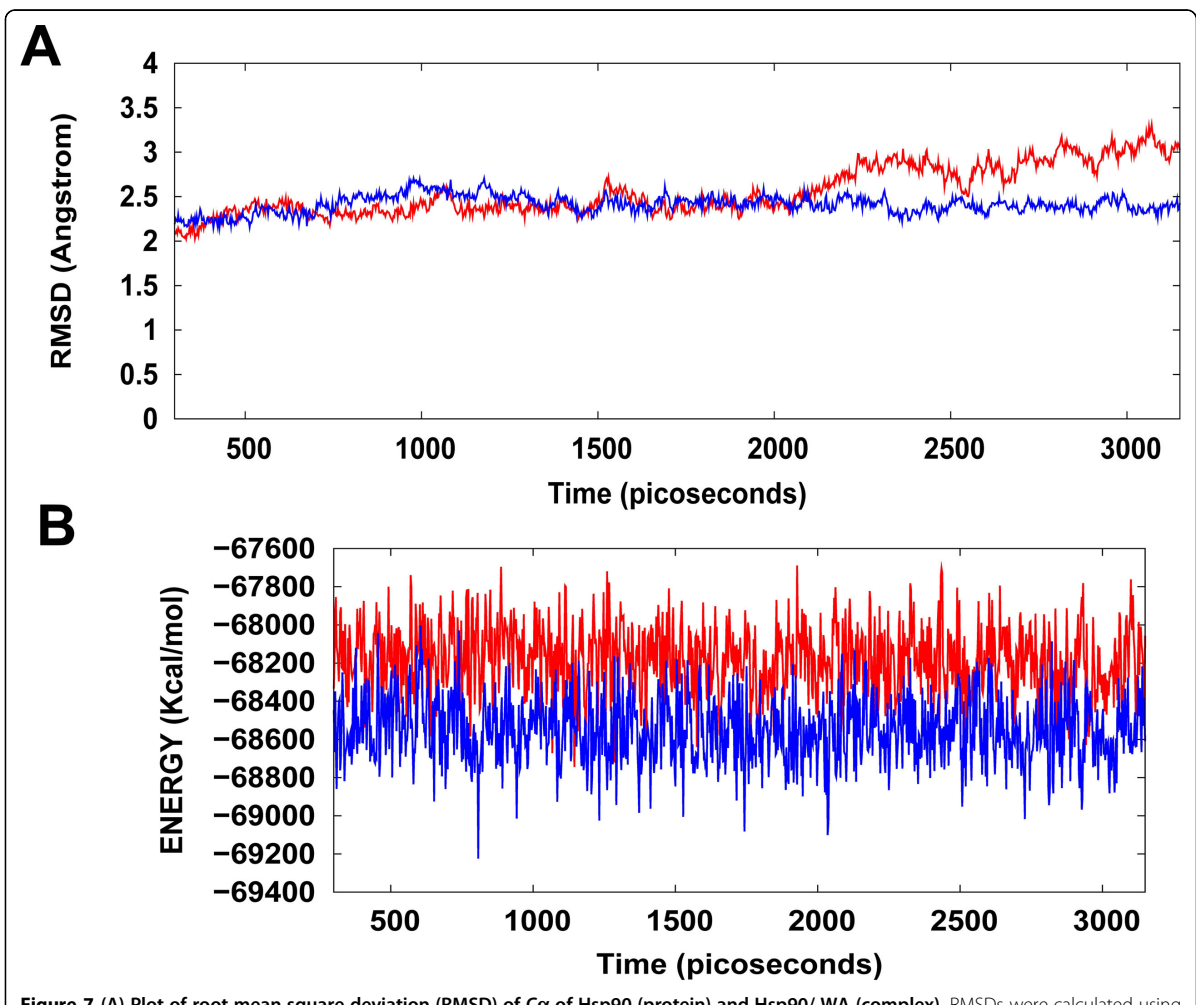
**(A) Plot of root mean square deviation (RMSD) of Cα of Hsp90 (protein) and Hsp90/ WA (complex).** RMSDs were calculated using the initial structures as templates. For protein (red) the reference is the PDB structure and for complex (blue) the reference is the initial docked structure. The trajectories were captured every 2.5 ps until the simulation time reached 3200 ps. **(B) Plot of total energy of Hsp90 and Hsp90/WA (complex)** The energy trajectories of both the protein (red) and the complex (blue) are stable over the entire length of simulation time.

The Hsp90/Cdc37/WA protein-drug binding complex with the binding energy of -9.68 kcal/mol obtained using ParDOCK was used for carrying out MD simulations. After the MD simulations, we calculated RMSDs between Cα of Hsp90/ Cdc37/WA complex trajectories recorded every 2.5 ps and Cα of Hsp90/Cdc37 association complex PDB structure. The RMSDs for the trajectories of Hsp90 complexed with WA were also calculated using the protein’s X-ray structure as a reference. The results in Figure 6A show that the RMSD trajectory of Hsp90/Cdc37/WA complex was less than 2.5 Å for the entire simulation and achieves a stationary phase in the later length of the simulation. It was noted that Hsp90/Cdc37/WA (complex) has an additional entity WA owing to which the RMSD of the complex is higher than the Hsp90/Cdc37 (protein) in the initial stage (0-700 ps) of simulation. However, as the simulation progresses, the ligand in association with the receptor protein tries to make interactions with the solvent molecules and thus stabilizes the associated structure; thus resulting in lowering of the RMSD trajectory in comparison to the protein. The trajectory for the total energy was also consistent throughout the simulation length. It was also found that the energy of the complex (blue) was lower than that of the protein (red) alone throughout the length of the simulation (Figure 6B), suggesting the stability of our docked protein-drug system. MD simulations were also carried out for Hsp90 in complex with WA. The consistency of RMSD and energy profiles of the complex of WA with Hsp90 (Figure 7) suggests the stability of WA even in complex with Hsp90 alone. However a larger binding energy obtained for Hsp90/Cdc37/WA complex along with the possibility of Hsp90/Cdc37 active complex formation even in WA complexed Hsp90 substantially merits the WA binding to the association complex in comparison to segregated Hsp90 binding. This supports the hypothesis that WA is quite a probable and a worth small molecule for targeting/inhibiting the chaperone/co-chaperone association complex. The simulation length used in this study was long enough to allow rearrangement of side chains of the native as well as the drug complexed receptors to find their most stable binding mode. In conclusion, the present MD simulations made clear the dynamic structural stability of Hsp90/Cdc37 in complex with the drug WA, together with the inhibitory mechanism. These results would be valuable for further designing non-covalent type inhibitors with high specificity and potent activity by alternatively targeting the Hsp90 signalling pathway in contrast to the classic Hsp90 inhibition involving blockage of ATP binding.

## Conclusions

The molecular chaperone Hsp90 has been a promising target for cancer therapy. Cancer is a disease marked by genetic instability. Thus specific inhibition of individual proteins or signalling pathways holds a great potential for subversion of this genetic plasticity of cancers. This study is a step forward in this direction. Our computational analysis provided a rationalization to the ability of naturally occurring WA to alter the chaperone signalling pathway. The large value of binding energy involved in binding of WA to the active Hsp90/Cdc37 complex consolidates the thermodynamic stability of the binding. Our docking results obtained substantiate the hypothesis that WA has the potential to inhibit the association of chaperone (Hsp90) to its co-chaperone (Cdc37) by disrupting the stability of attachment of Hsp90 to Cdc37. Conclusively our results strongly suggest that withaferin A is a potent anticancer agent as ascertained by its potent Hsp90-client modulating capability.

## Authors' contributions

AG, VSB and DS designed the methods and experimental setup. AG carried out the implementation of the various methods. AS, VA, PP and DB assisted AG in this process. AG and DS wrote the manuscript. All authors have read and approved the final manuscript.

## Competing interests

The authors declare that they have no competing interests.
